# Natural killer T cell sensitization during neonatal respiratory syncytial virus infection induces eosinophilic lung disease in re-infected adult mice

**DOI:** 10.1371/journal.pone.0176940

**Published:** 2017-06-01

**Authors:** Seung Young Lee, Youran Noh, Jung Hyun Goo, Semi Rho, Min Jung Kim, Chang-Yuil Kang, Manki Song, Jae-Ouk Kim

**Affiliations:** 1 Molecular Immunology Section, Clinical Research Lab, International Vaccine Institute, SNU Research Park, 1 Gwankak-ro, Gwanak-gu, Seoul, Korea; 2 College of Pharmacy, Seoul National University, Seoul, Korea; University of Tennessee Health Science Center, UNITED STATES

## Abstract

Respiratory syncytial virus (RSV) is a major viral pathogen that causes severe lower respiratory tract infections in infants and the elderly worldwide. Infants with severe RSV bronchiolitis tend to experience more wheezing and asthma in later childhood. Because invariant natural killer T (iNKT) cells are associated with the asthma pathology, we investigated whether neonatal iNKT cells are involved in the aggravation of pulmonary diseases following RSV infection in mice. Intranasal exposure to the iNKT cell ligand α-galactosylceramide (α-GC) with RSV primary infection in neonatal mice elicited neither cytokine production (except for a slight increase of IL-5) nor pulmonary eosinophilia, despite the presence of both CD1d^+^ cells and NKT cells. Interestingly, in adult mice re-infected with RSV, neonatal iNKT cell sensitization by α-GC during RSV primary infection resulted in much higher levels of pulmonary Th2 cytokines and elevated eosinophilia with airway hyperresponsiveness, whereas this was not observed in *cd1d* knockout mice. In contrast, α-GC priming of adults during RSV re-infection did not induce more severe airway symptoms than RSV re-infection in the absence of α-GC. α-GC co-administration during RSV primary infection facilitated RSV clearance regardless of age, but viral clearance following re-infection was not iNKT cell-dependent. This study clearly demonstrates that RSV-induced immune responses can be altered by iNKT cells, suggesting that neonatal iNKT cell sensitization during RSV primary infection is associated with exacerbation of pulmonary diseases following RSV re-infection in adulthood.

## Introduction

Respiratory syncytial virus (RSV) is a negative-sense single-stranded RNA virus. Most people are infected with RSV at least once by age 2 and then infected again later in life [1]. In healthy adults, RSV infection usually induces mild symptoms. However, in infants, elderly over the age of 65 years, and immunocompromised persons, RSV is a cause of morbidity and mortality associated with lower respiratory infection and bronchiolitis [1, 2]. RSV is estimated to cause 3.4 million hospitalizations and at least 66,000 deaths each year worldwide [3, 4].

Severe lower respiratory tract infection with RSV in infancy is considered an underlying cause of subsequent childhood asthma and wheezing [5–7]. Moreover, several studies implicate RSV as a cause of acute asthma exacerbation in both children and adults [8, 9]. Although the mechanisms linking early life RSV infection to subsequent asthma are not fully defined, some studies suggest that host susceptibility and immune factors play important roles [10, 11]. In humans, it is clear that Th2-skewed immunity prevails in newborns [12]. In neonatal mice, evidence indicates that Th1 immune responses against RSV are not induced by immature myeloid dendritic cells [13]. When mice are re-infected with RSV, Th2-skewed immune responses recur [14]. In mice, Th2-skewed immune responses to RSV at an early age may induce the development of an asthma-like phenotype on re-infection with the same virus [15–17].

Natural killer T (NKT) cells are a unique subset of lymphocytes that share properties of both T cells and natural killer (NK) cells [18, 19]. Most CD1d-dependent NKT cells, known as type I or invariant NKT (iNKT) cells, express a semi-invariant TCRα chain with a Vα14Jα18 gene segment in mice (Vα24Jα18 in humans), paired with a very limited TCRβ repertoire (Vβ8, 7, and 2 in mice and Vβ11 in humans). iNKT cells recognize glycolipid antigens such as α-galactosylceramide (α-GC) presented by the non-polymorphic major histocompatibility complex class I-like molecule, called CD1d, that is located on antigen-presenting cells [20]. The other type of CD1d-dependent NKT cells is called type II or non-invariant NKT cells. They do not express the Vα14Jα18 TCRα chain and do not recognize α-GC, but they do recognize other lipid antigens.

The ability of NKT cells to rapidly produce large amounts of cytokines enables this cell type to regulate a number of different inflammatory diseases, including infectious and autoimmune diseases, inflammatory bowel disease, cancer, and asthma [21]. Activation of NKT cells by microbes can lead to lung inflammation and airway hyperresponsiveness (AHR). For example, in mice infected with Sendai virus, NKT cells that produce IL-13 induce a chronic inflammatory process associated with AHR by promoting IL-13 production in alveolar macrophages [22]. Similarly, *Sphingomonas* bacteria are frequently identified in the lungs of patients with chronic asthma [23]. Those bacteria express glycolipids that directly activate NKT cells [24, 25]. Taken together, these studies suggest that NKT cells that are activated by some microbes in the lung play an important role in inflammation and AHR [21].

Because severe RSV infection and consequent iNKT immune responses can drive the development of asthma, we speculated that stimulation of iNKT cells during RSV infection in neonates might aggravate subsequent lung disease. In this study, we investigated the role of age-dependent iNKT cells in RSV infection-induced immunopathology in a mouse model. We found that neonatal iNKT cell sensitization during RSV primary infection is strongly associated with the exacerbation of eosinophilic lung diseases following RSV re-infection in adulthood.

## Materials and methods

### Ethics statement

This study was carried out in compliance with the Guide for the care and use of laboratory animals of National Institutes of Health and Korean national laws for animal welfare and laboratory animals. All mouse experimental procedures were approved by the Institutional Animal Care and Use Committee of International Vaccine Institute (2011–014 and 2014–012).

### Mice and welfare

Six-week-old female or 3-day-old BALB/c mice with their mother were purchased from Charles River Laboratories (Orient Bio, Seongnam, Korea) or Taconic (DaeHan Biolink [Chungbuk], Samtako Bio Korea, [Osan]). C.129S2-*Cd1*^*tm1Gru*^/J (CD1d knockout; CD1d KO) mice were purchased from Jackson Laboratory (Orient Bio). Mice were kept under specific pathogen-free conditions at Animal Research Facility in the International Vaccine Institute. Mice were provided with water and food *ad libitum*. All efforts were made to minimize suffering and the number of mice for research. The treated mice were monitored daily as part of the approved protocol. Until the end of the experiments, no mice died as a result of RSV infection. However, two mice died of unknown causes during i.n. administration of solutions under anesthesia. No mice met IVI's specific humane endpoint criteria for euthanasia (weight loss, decrease in appetite, weakness/inability to obtain feed or water, moribund state, unrelieved pain/distress, and organ dysfunction/failure etc.) during the experiments. At the end of each experimental time point, animals were sacrificed by cervical dislocation or CO_2_ inhalation.

### iNKT cell ligand and preparation of RSV stocks

A synthetic form of α-GalCer (KRN7000; Funakoshi, Tokyo, Japan; 1 mg/mL in 100% dimethyl sulfoxide, Sigma-Aldrich, St. Louis, MO) was used as a specific ligand for iNKT cells. A stock of RSV A2 strain (American Type Culture Collection [ATCC], Manassas, VA) was propagated in HEp-2 cells (ATCC) in 150-mm cell culture dishes. Four days after inoculation, virus was harvested and titer was determined by plaque assay. HEp-2 cells were maintained in minimum essential media (MEM) containing Earle’s salts, L-glutamine, 10% fetal bovine serum (FBS) (Hyclone, South Logan, UT), and 1% penicillin-streptomycin (Gibco, Grand Island, NY).

### RSV infections

One-week-old neonatal BALB/c mice were anesthetized by intraperitoneal injections with ketamine hydrochloride (Yuhan, Seoul, Korea; 0.1 mg/g bodyweight) combined with xylazine hydrochloride (Rompun; Bayer Korea, Seoul; 12.5 μg/g bodyweight) and intranasally (i.n.) administered one of four different 5-μL solutions: live RSV A2 (5 × 10^5^ PFU), α-GC (1 μg), live RSV A2 (5 × 10^5^ PFU) with α-GC (1 μg), or vehicle alone (vehicle: dimethyl sulfoxide 1 μL and PBS 4 μL). Six-week-old mice were also i.n. administered one of four different solutions in 20 μL: live RSV A2 (1 × 10^6^ PFU), α-GC (1 μg), live RSV A2 (1 × 10^6^ PFU) with α-GC (1 μg), or vehicle (PBS).

To test the effects of neonatal iNKT cells, 1-week-old neonatal BALB/c or CD1d KO mice were i.n. administered 5 μL of live RSV A2 (5 × 10^5^ PFU), α-GC (1 μg), live RSV A2 (5 × 10^5^ PFU) with α-GC (1 μg), or vehicle (PBS). At 8 weeks of age, all mice were challenged with live RSV A2 (1 × 10^6^ PFU/20 μL).

### Analysis of cells and cytokines in bronchoalveolar lavage (BAL) fluid

Four days after challenge with RSV A2, mice were sacrificed, and adult and neonate tracheas were cannulated and washed with 700 and 300 μL of PBS, respectively. After BAL fluid was centrifuged, supernatant was stored at -80°C until analysis. Cells were incubated with violet fluorescent live-dead discriminator (Invitrogen, Eugene, OR) for 10 min at room temperature and then washed with 1 mL of PBS and blocked for 5 min with purified CD16/CD32 Fc (clone 2.4G2; BD Pharmingen, San Jose, CA). After blocking, 50 μL of antibody cocktail containing anti-CD45-APC (clone 30-F11), CD11c-FITC (clone HL3), Ly-6G (Gr-1)-PE-Cy7 (clone: 1A8), and Siglec F-PE (clone E50-2440; all from BD Pharmingen) were added to cells and incubated at 4°C for 30 min. Cells were subsequently washed two times with PBS (2% FBS) and fixed with 200 μL of paraformaldehyde. Cells were analyzed by using a BD FACS LSR II flow cytometer and data were analyzed with FlowJo software (version 10; Tree Star, Ashland, OR). The cytokines in BAL supernatant was measured using the mouse Th1/Th2/Th17 BD Cytometric Bead Assay Kit (BD Biosciences, San Jose, CA) according to the manufacturer’s recommendations. The cytokine levels were also analyzed using the Mouse Magnetic Luminex Screening Assay (R&D, Minneapolis, MN) [26].

### Analysis of cells in lung

Four days after RSV A2 challenge, neonatal and adult mice were sacrificed and their lungs harvested. Lungs were chopped and incubated with lung enzyme cocktail including 10% RPMI (Sigma, St. Louis, MO), collagenase D (Roche, Mannheim, Germany), and DNase1 (Roche) for 30 min at 37°C with stirring. After incubation, lung homogenates were transferred to a 70-μm strainer on a 50-mL conical tube and cells were analyzed by flow cytometry as described above. Anti-CD1d-APC (clone 1B1; eBioscience, San Diego, CA), CD3ε-PerCP-Cy5.5 (clone 145-2C11; BD Pharmingen), and CD49b-PE (clone DX5; BD Pharmingen) were used for lung cell staining.

### Lung RSV detection

Lung tissues were washed by vascular perfusion with PBS containing heparin (10 U/mL) and then homogenized by passing through a 70-μm cell strainer (BD Labware, Franklin Lakes, NJ) with 2 mL of MEM (10% FBS). Lung homogenates were centrifuged at 300 ×g for 5 min and 100 μL of supernatants was inoculated into 90% confluent HEp-2 cells in 6-well plates. After incubation with an agar overlay for 5 days, each well was stained with 0.1% crystal violet before plaques were counted to determine the PFU/mL.

### Lung histology

Four days after RSV A2 challenge, lungs were isolated and fixed with 4% formalin. Formalin-preserved lungs were embedded in paraffin, sectioned into 5-μm thicknesses, and stained with hematoxylin-eosin (H&E) or periodic acid–Schiff (PAS).

### Measurement of airway responsiveness

The methacholine challenge test was used to evaluate AHR, a hallmark of asthma. Four days after RSV challenge, mice received nebulized methacholine (0 and 10 mg/mL) for 3 min and enhanced pause (Penh) was recorded for 3 min using whole-body plethysmography (OCP 3000, Allmedicus, Korea). Penh at 10 mg/mL was expressed as the value obtained from each mouse subtracted from the value for the nebulized PBS inhalation control (0 mg/mL).

### Statistical analysis

Data were analyzed using Prism software (version 5; GraphPad, La Jolla, CA) and expressed as mean ± SEM. Statistical significance was determined by using an unpaired, two-tailed Student *t* test. *P* values less than 0.05 were considered statistically significant.

## Results

### α-GC does not cause eosinophil recruitment or induce cytokine secretion in neonatal mice but does in adult mice

**Fig 1:**
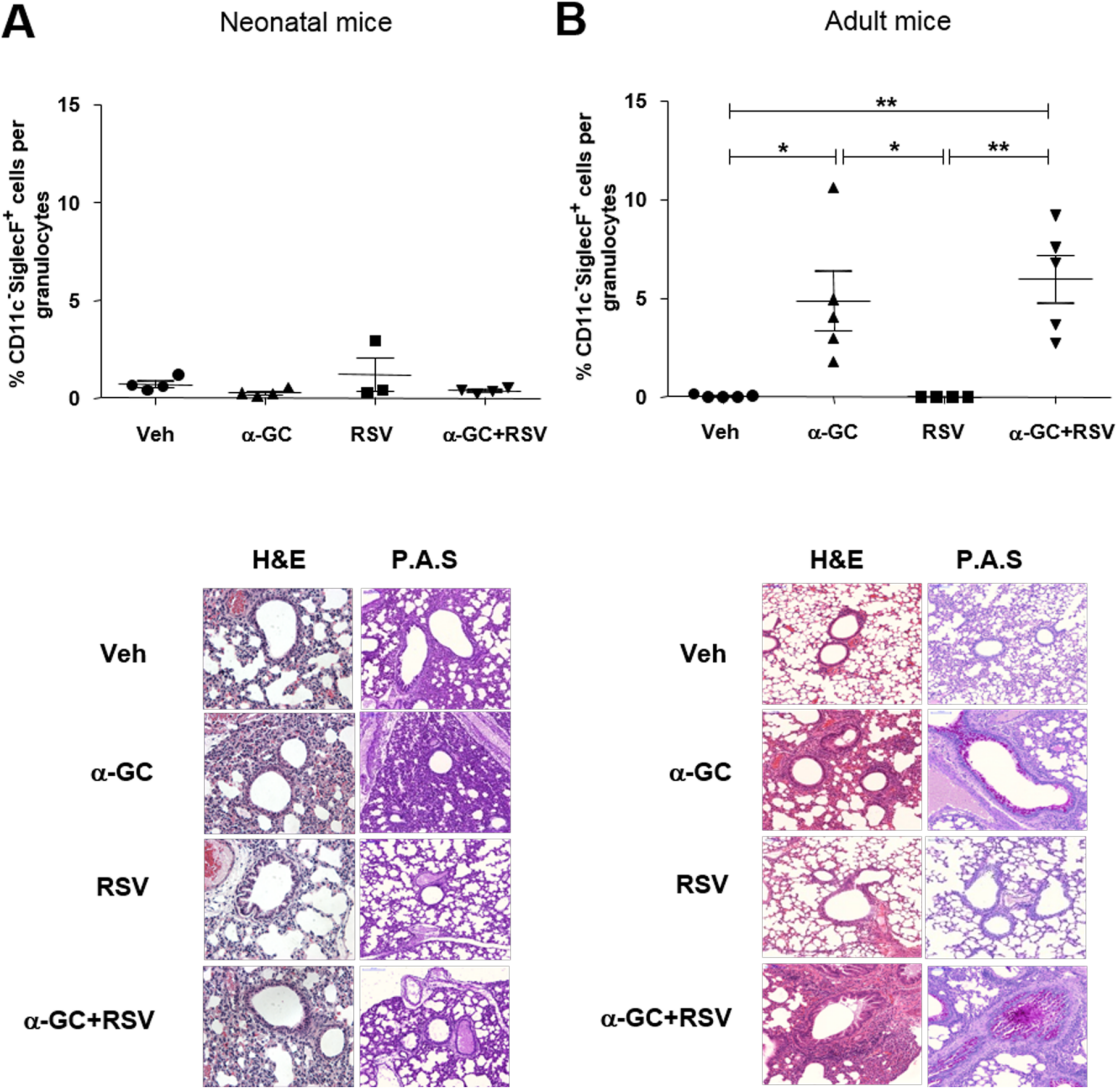
Pulmonary eosinophil infiltration and mucus production after NKT cell ligand administration in neonatal and adult mice. Mice were i.n. administered vehicle (Veh), α-GC, RSV, or α-GC+RSV at ages 7 days (neonates) (A) and 6 weeks (adults) (B). At 4 days after injection, cells were isolated from BAL fluid and stained with fluorescence-labeled anti-CD45, CD11c, and Siglec-F antibodies. Percentages of eosinophils, namely CD11c^-^SiglecF^+^ cells, among granulocytes are shown. All data are mean ± SEM. (A) Data are for 6–8 mice per group using pooled samples from 2 neonatal mice and are representative of 4 independent experiments. *p<0.05; **p<0.01 (B) Data are for 4 or 5 mice per group and are representative of 2 independent experiments. Lung tissues in left and right panels, respectively, are stained with hematoxylin-eosin (H&E) and periodic acid–Schiff (PAS).

**Fig 2:**
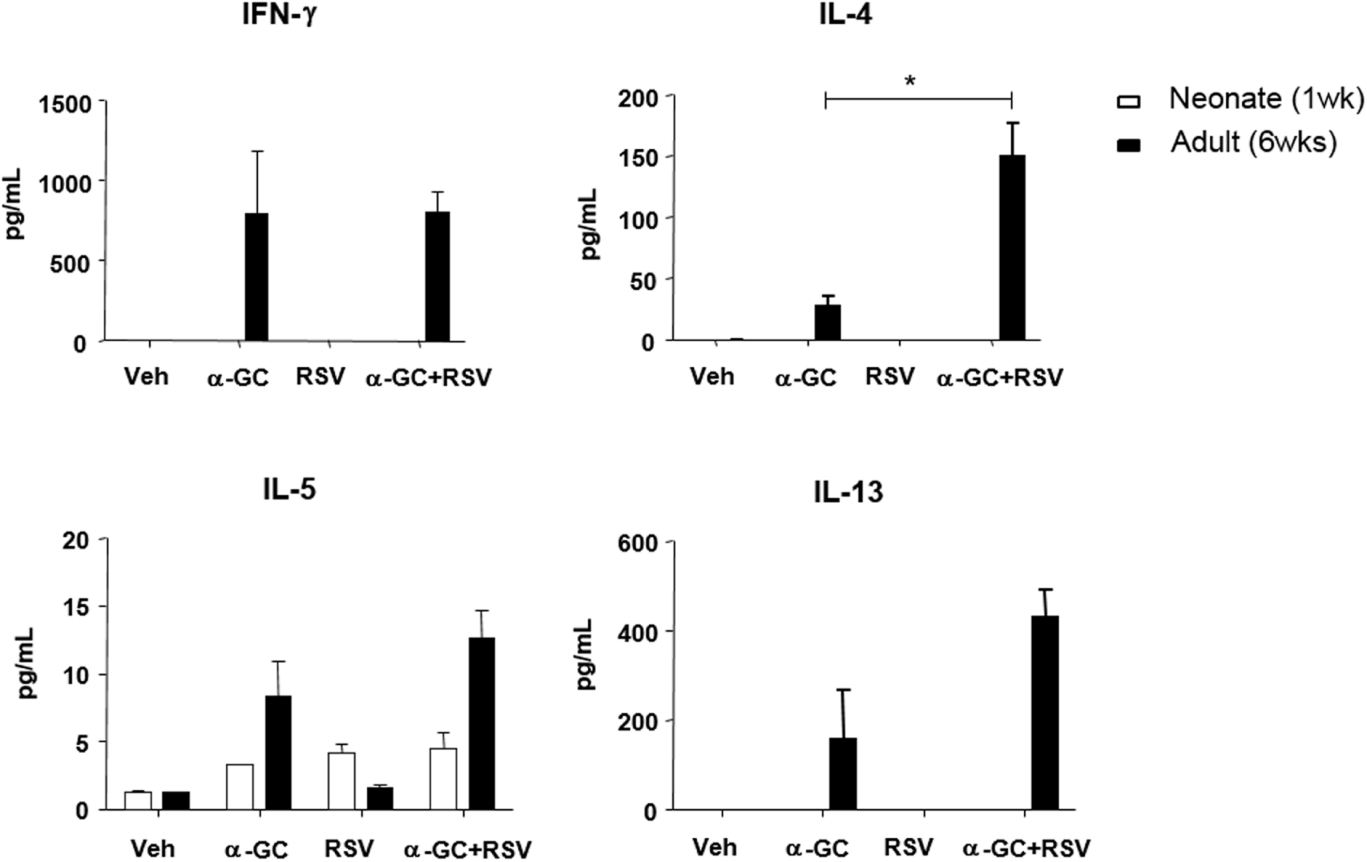
Cytokine production in BAL fluid in response to RSV infection with or without α-GC. (A) Mice were i.n. administered vehicle (Veh), α-GC, RSV, or α-GC+RSV at ages 7 days and 6 weeks. BAL fluid was collected from lungs 4 days after infection. Cytokine levels in BAL fluid were determined by luminex and cytometric bead assay. Data are expressed as mean ± SEM of 9–12 mice in each group of neonates and of 3 or 4 mice in each group of adults. *p<0.05

We investigated the effect of iNKT cells on RSV-induced immune responses by age. First, neonatal (7-day-old) or adult (6-week-old) BALB/c wild-type mice were i.n. administered with vehicle (dimethyl sulfoxide and phosphate buffer saline), α-GC, RSV, or α-GC+RSV. Four days later, we examined eosinophil infiltration in BAL fluid. In adult mice, eosinophils were found in BAL fluid in the α-GC and α-GC+RSV treatment groups (Fig 1B, S1B Fig); however, α-GC and RSV had no effect on the recruitment of eosinophils in BAL fluid of neonatal mice (Fig 1A, S1A Fig). Eosinophil recruitment corresponded with H&E staining of lungs (Fig 1A and 1B). In adults, cell infiltration and mucus production were observed in both the α-GC and α-GC+RSV treatment groups (Fig 1B), but no neonate treatment group showed additional cell infiltration or mucus production in lungs (Fig 1A). Administration with neither α-GC nor RSV affected production of pulmonary cytokines (e.g., IFN-γ, IL-4, and IL-13) in neonates. There was a slight increase of IL-5, but the difference was not significant between the α-GC, RSV, and α-GC+RSV groups. In contrast, in adults, both α-GC and α-GC+RSV increased IFN- γ, IL-4, IL-5, and IL-13 levels (Fig 2). In adult mice, α-GC+RSV administration elevated IL-4 production more than administration of α-GC alone (p < 0.05). IL-17 was below the detection level of the assay in all conditions. Collectively, these results show that in neonatal mice neither α-GC nor RSV affects eosinophil recruitment, mucus production, or cytokine production, with the exception of IL-5.

### Neonatal NKT cells are unresponsive to α-GC administration despite the presence of CD1d-positive cells

**Fig 3:**
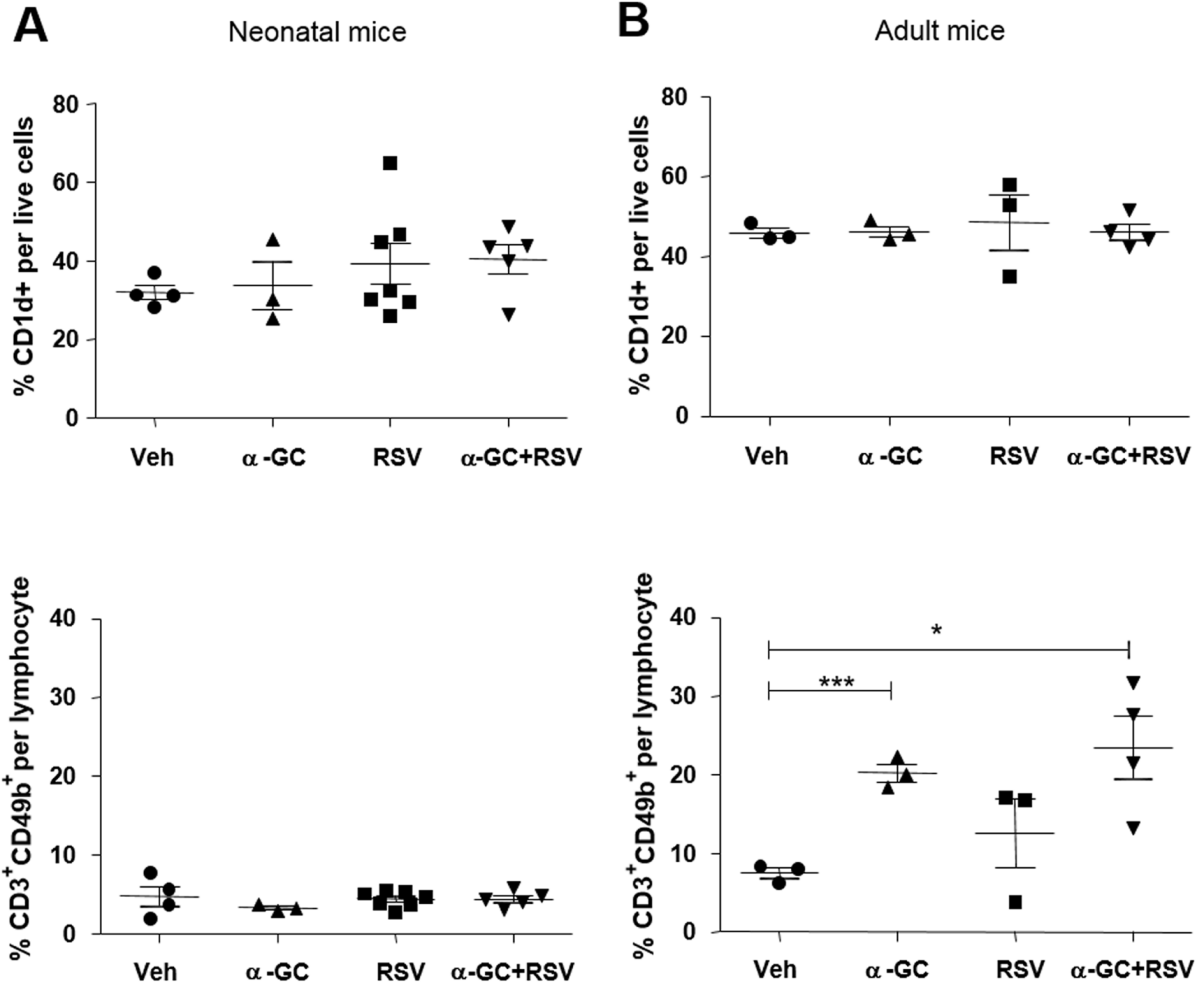
CD1d expressing and NKT cells in neonatal and adult mice. (A) Mice were i.n. administered vehicle, α-GC, RSV, or α-GC+RSV at ages 7 days (A) and 6 weeks (B). After 4 days, lungs were harvested and stained with fluorescence-labeled anti-CD1d, CD3ε, and CD49b antibodies. Percentages of CD1d-positive or NKT cells (CD3^+^CD49b^+^) are shown. Data are shown as mean ± SEM with 3–7 mice per group (A) and with 3 or 4 mice per group (B). *p<0.05; ***p<0.001.

We assumed that unresponsiveness of neonatal iNKT cells to i.n. administration of α-GC might result from the lack of CD1d-expressing cells, which present α-GC to iNKT cells; however, both neonatal (Fig 3A, S2A Fig) and adult (Fig 3B, S2B Fig) mice had similar ratios and absolute numbers of CD1d-expressing cell populations. In addition, CD3^+^CD49b^+^ NKT cells exist in neonatal mice, although in our study, the ratio of neonatal NKT cells remained consistent, regardless of α-GC or RSV administration (Fig 3A, S2A Fig). In contrast, in adult mice, the ratio and absolute numbers of NKT cells increased after α-GC administration (p < 0.001 for vehicle vs. α-GC; p < 0.05 for vehicle vs. α-GC+RSV) (Fig 3B). Collectively, these results suggest that neonatal mice have sufficient numbers of CD1d-expressing cells; however, unlike adult mice, they lack the ability to increase their NKT cell ratios, recruit eosinophils, and produce cytokines in response to α-GC.

### Co-administration of α-GC with RSV primary infection enhances CD1d-mediated viral clearance

**Fig 4:**
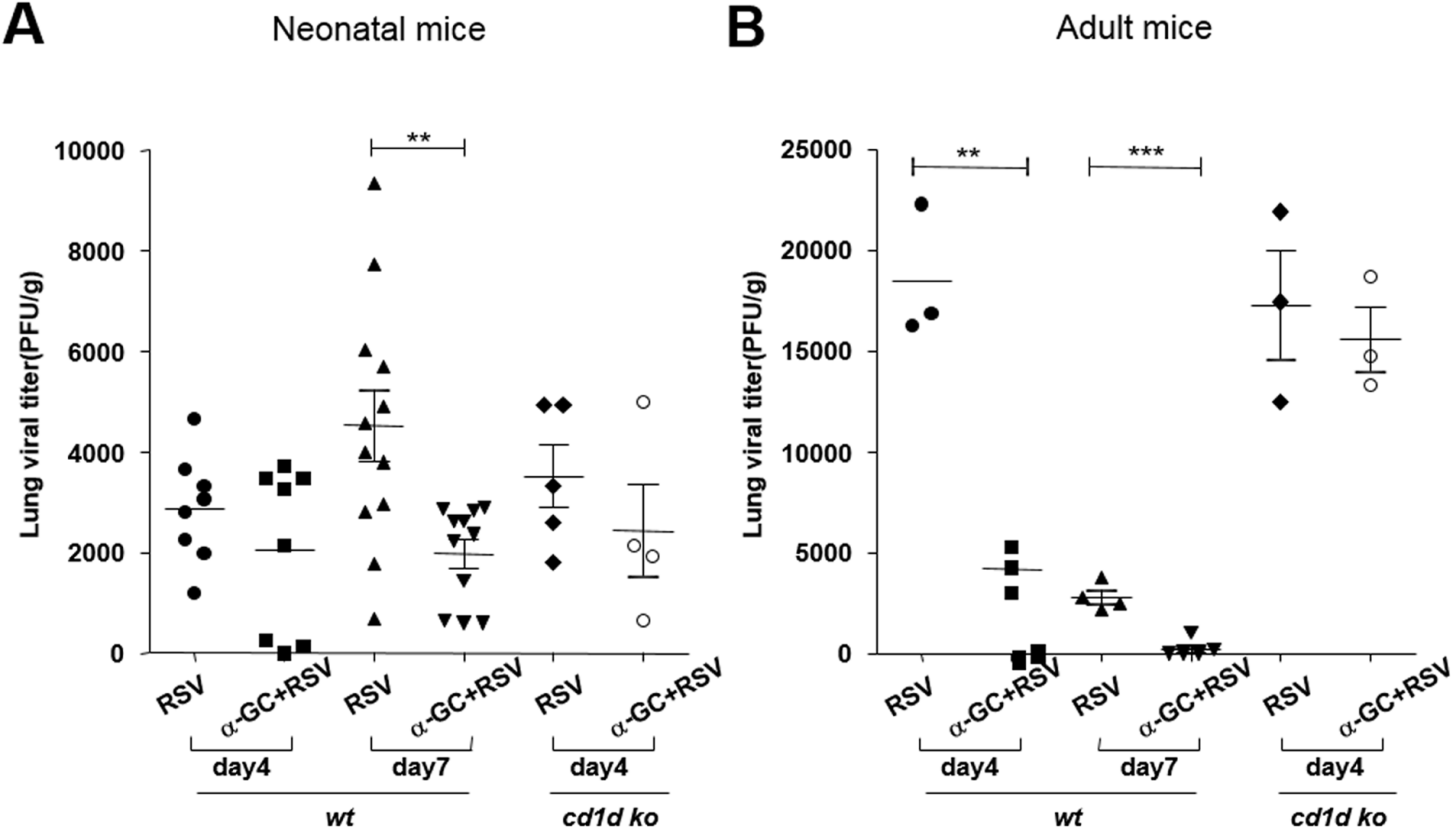
RSV clearance by intranasal (i.n.) α-GC co-administration at neonate or adult ages. BALB/c or CD1d KO mice were infected i.n. with RSV or α-GC+RSV at ages 7 days (neonates; A) or 6 weeks (adults; B). On day 4 or 7 after administration, lungs were harvested and lung homogenates were assessed by plaque assay. Data are shown as mean ± SEM with 4–8 mice per group (A) and 3–5 mice per group (B). **p<0.01; ***p<0.001; PFU, plaque forming units.

Because α-GC can stimulate iNKT cells, which are part of the innate immune system, we next investigated whether co-exposure to α-GC and RSV infection enhanced RSV clearance. Mice were infected with RSV or α-GC+RSV, and virus titers were analyzed at 4 or 7 days after infection. In neonatal mice, α-GC with RSV had a statistically significant effect on virus clearance from lungs at 7 days after infection (p < 0.01) (Fig 4A), but not at 4 days. In contrast, in adult mice, co-administration of α-GC with RSV enhanced clearance of virus from lungs at both 4 days (p < 0.01) and 7 days (p < 0.001) after infection (Fig 4B). To test whether the effects of α-GC were mediated by CD1d, CD1d KO mice were infected with RSV or α-GC+RSV. When analyzed 4 days after infection, viral titers showed that clearance of virus in response to α-GC was dependent on the CD1d molecule (Fig 4A and 4B). Collectively, these results suggest that administration of α-GC can enhance the clearance of RSV from lungs, regardless of age, and that its effects are dependent on CD1d, although clearance of RSV in conjunction with α-GC administration is more potent and faster in adults than in neonatal mice.

### iNKT cell ligand co-exposure during RSV primary infection in neonatal mice elevates adult immune responses after RSV re-infection

**Fig 5:**
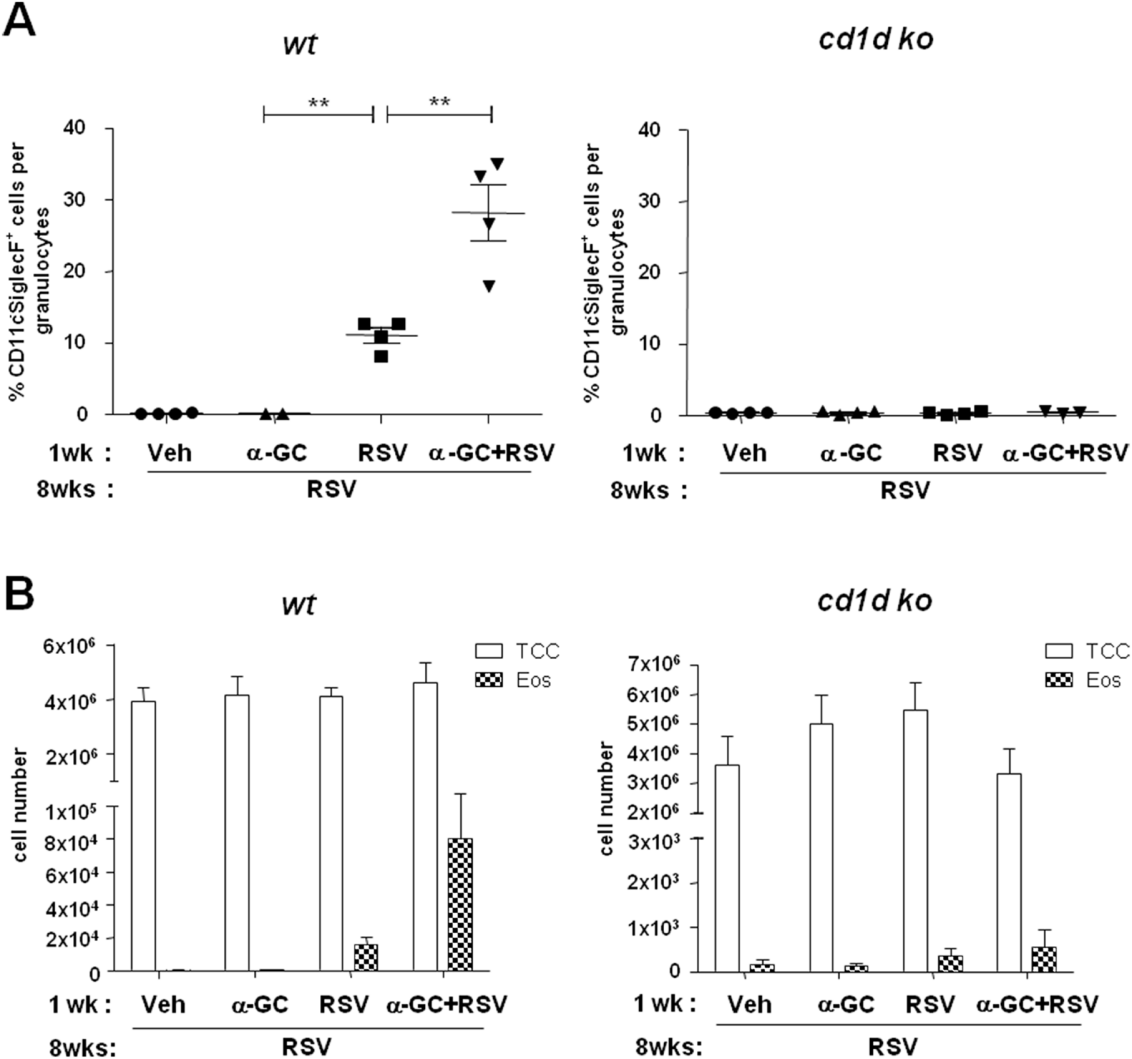
Effects of neonatal α-GC sensitization with RSV primary infection on eosinophils following re-infection in adulthood. Wild-type or CD1d KO neonatal mice were i.n. administered PBS, α-GC, RSV, or α-GC+ RSV and challenged at 8 weeks of age with RSV. Four days after challenge, cells were isolated from the BAL fluid, and the ratio of eosinophils to granulocytes was determined as shown in Fig 1 (A). The total cell count and absolute number of eosinophils are shown (B). Data are mean ± SEM with 2–4 mice per group and representative of three independent experiments. **p<0.01.

**Fig 6:**
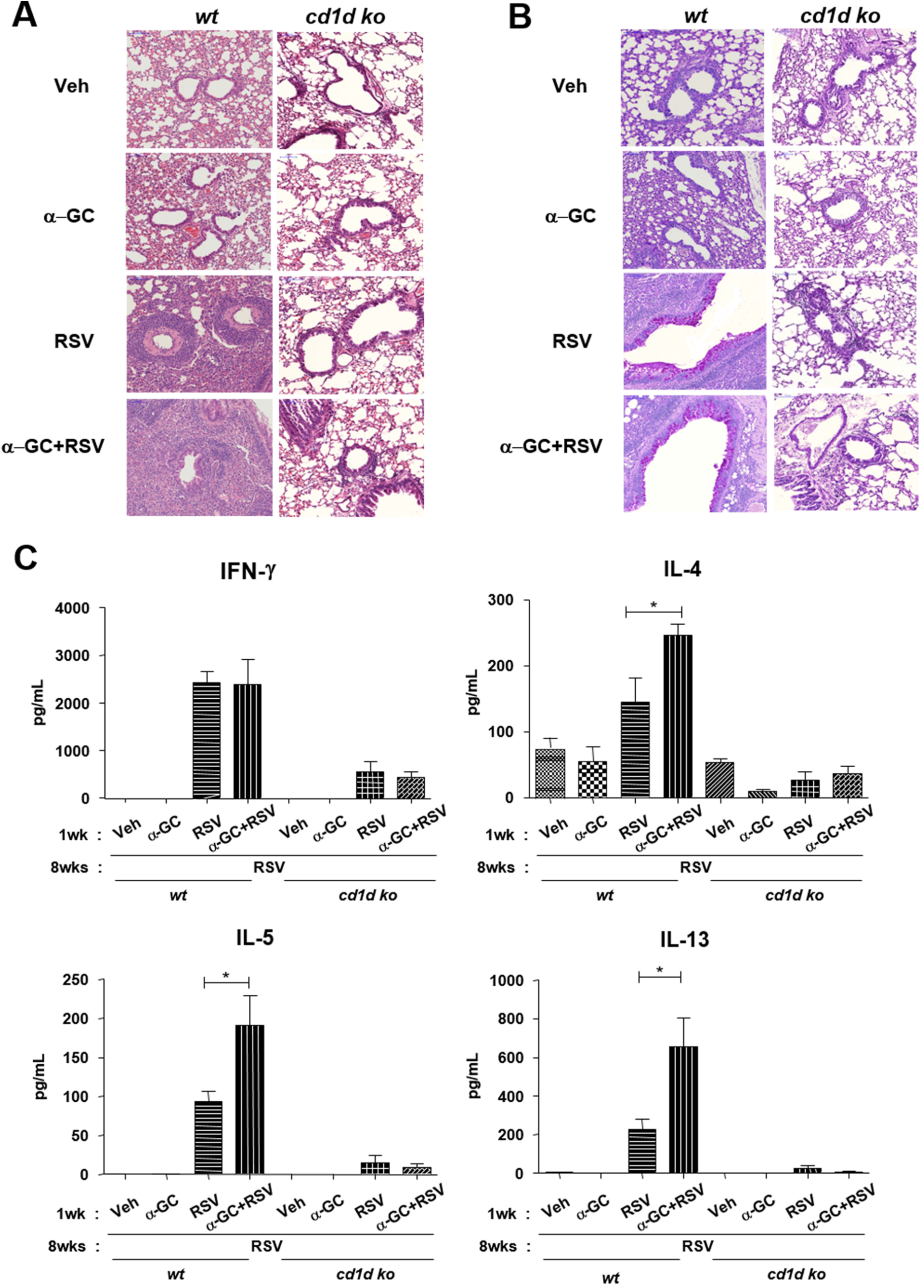
Effects of neonatal α-GC sensitization with RSV primary infection on pulmonary immune responses following re-infection in adulthood. Wild-type or CD1d KO neonatal mice were i.n. administered PBS, α-GC, RSV, or α-GC+ RSV and challenged at 8 weeks of age with RSV. Lung tissue was formalin-fixed, paraffin-embedded, and stained with hematoxylin-eosin (H&E) (A) or periodic acid–Schiff (PAS) (B) on day4 after RSVA2 challenge. (C) Cytokine levels in BAL fluid were determined by luminex assay. Data are expressed as mean ± SEM of 5 or 6 mice per group. *p<0.05.

We further examined whether neonatal NKT cell sensitization by α-GC affects immune responses in adults following RSV re-infection. Neonatal mice were administered vehicle, α-GC, RSV, or α-GC+RSV, and challenged with RSV at 8 weeks of age. There were increased numbers of eosinophils in the BAL fluid of groups exposed to RSV or α-GC+RSV in wild-type neonates. The number of infiltrated eosinophils was highest in the α-GC+RSV group. However, this pulmonary eosinophilia was abrogated in CD1d KO mice, suggesting that eosinophil infiltration following RSV re-infection is CD1d-dependent (Fig 5). In addition, lung histology showed similar CD1d-mediated cell recruitment (Fig 6A) and mucus production (Fig 6B) in both RSV and α-GC+RSV treatment groups. Th2 cytokines such as IL-4, IL-5, and IL-13 were increased most when mice were administered α-GC+RSV as neonates; IFN-γ levels were comparable in the RSV and α-GC+RSV groups (Fig 6C). CD1d KO mice produced much lower levels of cytokines than wild-type mice, and the differences between the RSV and α-GC+RSV groups were not statistically significant (Fig 6C).

**Fig 7:**
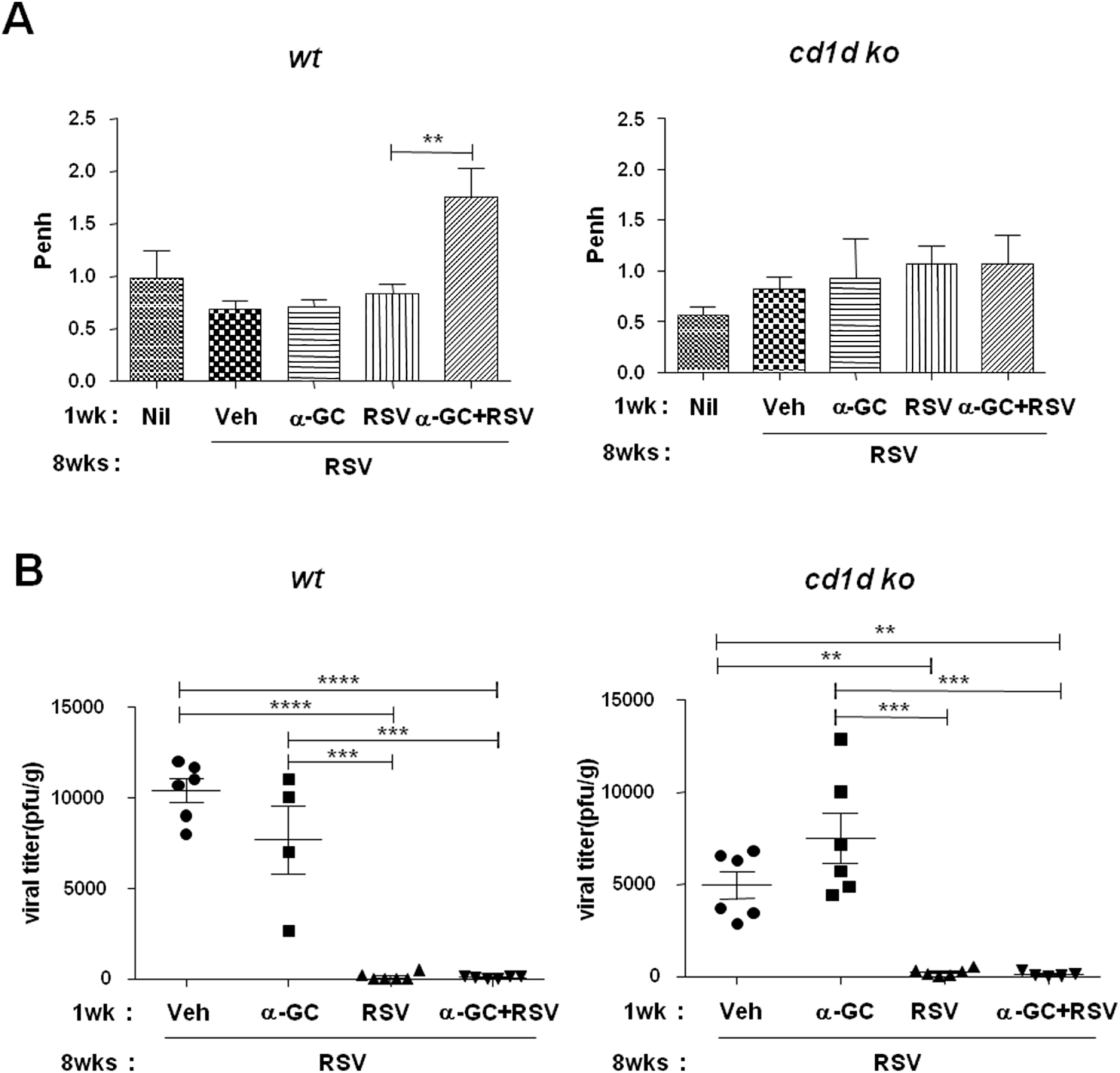
Effects of neonatal α-GC sensitization with RSV primary infection on airway hyperresponsiveness and viral clearance following re-infection in adulthood. (A) Airway hyperresponsiveness measured by methacholine test (Penh). Wild-type (wt) or CD1d knockout neonatal mice were infected with vehicle (Veh), α-GC, RSV, or α-GC with RSV and at age 8 weeks they were challenged with RSV. Four days after challenge, mice were given nebulized methacholine; 0 or 10 mg/mL. Data are expressed as mean ± SEM of 6 mice given 10 mg/mL of methacholine. **p<0.01. (B) Lungs were harvested and viral titers assessed by plaque assay on day 4 after challenge. Results are representative of two independent experiments. Data are expressed as mean ± SEM of 4–6 mice per group. **p<0.01; ***p<0.001; ****p<0.0001.

We next examined whether elevated lung eosinophil infiltration and Th2 cytokine production were correlated with severe AHR. Methacholine testing 4 days after RSV re-infection showed that α-GC priming during RSV primary infection led to more severe AHR in neonatal mice than priming with RSV alone (Fig 7A). Severe AHR was not detected in the CD1d KO mice (Fig 7A). In contrast, when wild-type and CD1d KO mice were re-infected with RSV in the absence of α-GC, the virus was cleared (Fig 7B). These results suggest that RSV itself elicited CD1d-independent acquired immune protection against RSV re-infection.

Collectively, these results suggest that neonatal sensitization of iNKT cells by α-GC does not immediately induce an immune response, but that sensitized NKT cells elevate eosinophil recruitment, Th2 cytokine production, and AHR following RSV re-infection in adulthood. However, NKT cell sensitization in neonatal mice does not affect lung viral clearance following RSV re-infection in adulthood.

### Adult NKT cell stimulation during RSV re-infection does not aggravate pulmonary eosinophilia

**Fig 8:**
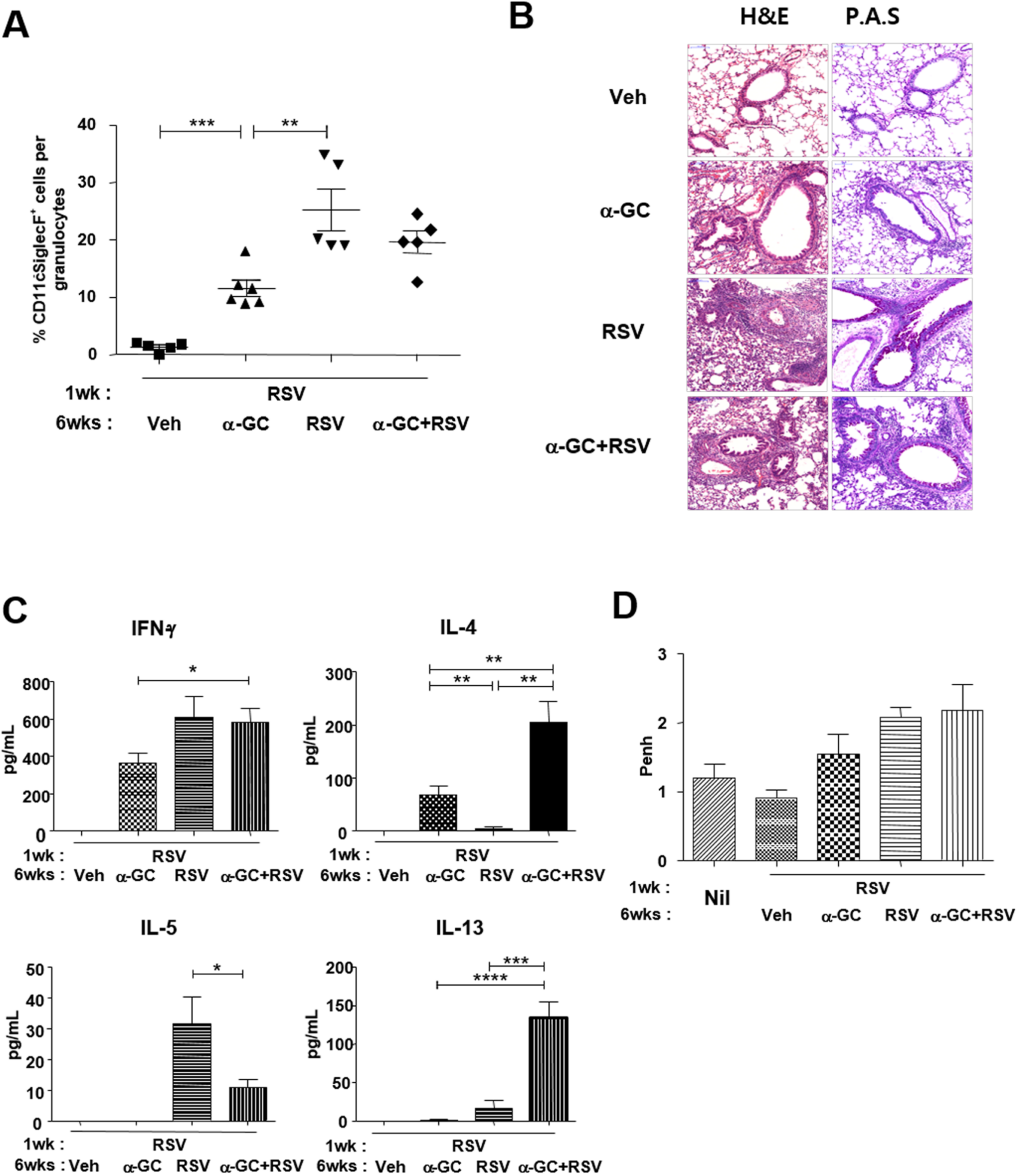
Effects of adult NKT cell priming by α-GC during RSV re-infection. Neonate mice (7 days old) were infected with RSV and at age 6 weeks i.n. administered vehicle (Veh), α-GC, RSV, or α-GC with RSV. (A) Four days after infection, cells were isolated from BAL fluid and the ratio of eosinophils per granulocyte determined as described in Fig 1. Data are mean ± SEM with 5 or 6 mice per group. **p<0.01; ***p<0.001. (B) Histologic examination of lungs on day 4 after infection. Lung tissue was formalin-fixed, paraffin-embedded, and stained with hematoxylin-eosin (H&E) or periodic acid–Schiff (PAS). (C) Cytokine levels in BAL fluid were determined by luminex assay. Data are mean ± SEM of 5 or 6 mice per group. *p<0.05; **p<0.01; ***p<0.001; ****p<0.0001. (D) Airway hyperresponsiveness was measured by methacholine test (Penh) 4 days after infection when mice were given nebulized methacholine; 0 and 10 mg/mL. Data are expressed as mean ± SEM of 5 or 6 mice at 10 mg/mL of methacholine in each group.

To examine whether adult iNKT cell stimulation during RSV re-infection also plays a role in the exacerbation of lung disease, wild-type mice were re-infected with RSV in the presence of α-GC following neonatal primary infection. The RSV re-infection groups showed significantly higher levels of eosinophils in BAL fluid than the group of neonates primed with RSV but administered α-GC only in adulthood (p < 0.01, RSV vs. α-GC). There was no significant difference in pulmonary eosinophilia between the two groups of mice re-infected with RSV, regardless of whether α-GC was co-administered (Fig 8A, S3 Fig). Histology data also showed similar levels of cell infiltration and mucus production after RSV re-infection, regardless of α-GC priming in adulthood (Fig 8B). Similarly, the higher IFN-γ levels in BAL fluid were comparable in the two groups, whether or not α-GC was co-administered, but IL-4 and IL-13 levels were much higher in the group stimulated with α-GC during RSV re-infection. Of interest, α-GC priming during RSV re-infection led to decreased levels of IL-5, relative to levels in mice re-infected without α-GC (Fig 8C). AHR as a direct marker of asthma was not significantly different between the RSV and α-GC+RSV groups (Fig 8D). This suggests that adult iNKT cell stimulation during RSV re-infection does not exacerbate eosinophilic lung disease.

## Discussion

Our results show that neonatal iNKT cell sensitization during RSV primary infection can promote immune responses in lungs upon RSV re-infection. This may be associated with the development of asthma following RSV re-infection, contrary to adult iNKT cell stimulation during RSV re-infection.

One study reported that the age of first RSV infection is critical for determining cytokine production and disease patterns during adult RSV re-infection [15]. Neonates tend to display Th2-biased responses with prolonged memory, whereas Th1 memory is unstable [27–29]. Although neonatal exposure to α-GC with RSV does not appear to induce an immediate response, our results suggest there may be prolonged Th2-biased memory. Therefore, during RSV re-infection in adult mice, iNKT cells sensitized in neonates promoted the recruitment of eosinophils to lungs and secretion of Th2-type cytokines (Fig 5). We found no difference in viral clearance after re-infection, perhaps because adaptive immunity is sufficient for clearance during RSV re-infection.

Eosinophilic inflammation of airways, which includes an increase in activated and degranulated eosinophils, is the key feature of both allergic and non-allergic asthma [30, 31]. There are significant correlations between the activation of eosinophils and the severity of asthma as reflected in bronchial hyperresponsiveness and asthma symptom scores [32]. Th2 cytokines such as IL-4, -5, and -13 are considered markers of asthma, and IL-4 is the main cytokine involved in the pathogenesis of allergic disorders, including stimulation of mucus-producing cells and fibroblasts [33, 34]. Large amounts of IL-4 can lead to lymphocytic and eosinophilic inflammation, but without airway hyperreactivity [35, 36]. IL-13 is closely related to IL-4, which binds to IL-4R receptors and is also expressed by Th2 cells from asthma patients [37]. Increased amounts of IL-13 are observed in the airways of patients with atopic and non-atopic asthma [38, 39]. IL-5 is highly specific for eosinophilic inflammation and may play an important role in eosinophil survival, maturation, and activation in asthma [40, 41]. The increased percentage of eosinophils in sputum and elevated AHR in asthma are correlated with IL-5 secretion [42, 43]. In addition, inhibition of IL-5 was shown to be effective in reducing eosinophilic inflammation and AHR in various species [40].

NKT cells can be immediately activated and rapidly produce large amounts of cytokines such as IL-4, IL-5, IL-13, IL-17, and IFN-γ in response to glycolipid antigens such as α-GC [21, 44–46]. During this process, NKT cells also interact with other cells of the immune system, including eosinophils, and lead cell activation, recruitment, and differentiation [47]. Activated NKT cells secrete IL-5, which recruits eosinophils directly to the lung, or secrete IL-4 and IL-13, which causes lung epithelial and endothelial cells and lung fibroblasts to secrete eotaxin. In response, eosinophils are recruited to the lung [48].

In neonatal mouse iNKT cells stimulated by α-GC, enhanced Th2 cytokine production and eosinophil infiltration in lungs after RSV re-infection seem to exacerbate AHR compared to primary infection with RSV alone. However, stimulation of iNKT cells with α-GC alone did not lead to airway inflammation in response to sequential RSV infection. These results suggest that RSV-specific adaptive immune responses are indispensable for the development of this pathology.

Priming of adult iNKT cells with α-GC during RSV re-infection elicits immune responses that differ from those associated with neonatal iNKT cell priming. iNKT cells stimulated with α-GC during RSV re-infection resulted in the secretion of more IL-4 and IL-13 in lungs than that found in mice re-infected with RSV alone. In our study, iNKT cell priming with RSV re-infection of adult mice did not promote recruitment of airway eosinophils or AHR with decreased IL-5. These results show that adult iNKT cell stimulation during RSV re-infection does not aggravate eosinophilic lung disease. The number of infiltrated neutrophils (CD11b^+^CD11c^-^Gr-1^+^) was increased in RSV re-infected adult mice, regardless of α-GC exposure during neonatal primary infection or re-infection (S4 Fig). In addition, IL-17 was below the detection level of the assay in our experimental conditions.

Collectively, our results show that neonatal iNKT cell sensitization is involved in exacerbation of lung inflammation and asthma pathology following RSV re-infection. RSV re-infection without α-GC co-administration also led to CD1d-mediated airway inflammation, suggesting that natural RSV infection results in CD1d-dependent NKT cell stimulation. If so, RSV itself may have NKT cell ligands or may promote presentation of natural ligands to NKT cells via CD1d-expressing cells in vivo.

Our results suggest that stimulation of iNKT cells during neonatal RSV infection may be a cause of asthma aggravation following later RSV re-infection.

## Supporting information

S1 Fig Pulmonary eosinophils infiltration after NKT cell ligand administration in neonatal/adult mice. Mice were administered vehicle, α-GC, RSV, or α-GC+RSV via intranasal route when they were 7 days old (neonatal mice) (A) and 6 weeks old (adult mice) (B). 4 days after administration, cells were isolated from BAL fluids and stained with fluorescence-labeled anti-CD45, CD11c, and Siglec-F antibodies. Total cell count and the absolute number of eosinophils, namely CD11c-SiglecF+ cells, was shown. Data are shown as mean±SEM with n = 6–8 mice per group using pooled samples from 3 neonatal mice and representative of 4 independent experiments (A). Data are shown as mean±SEM with n = 4–5 mice per group (individual) and representative of 2 independent experiments (B).
(TIF)

S2 Fig Expression of CD1d in neonatal/adult mice. (A) Mice were infected with vehicle, α-GC, RSV, or α-GC+RSV via intranasal route when they were 7 days old (neonatal mice) (A) and 6 weeks old (adult mice) (B). After 4days, lungs were harvested and stained with fluorescence-labeled anti-CD1d, CD3 and CD49b^+^ antibodies. Total cell count and the absolute number of CD1d positive cell, or NKT cells (CD3^+^ CD49b^+^) were shown. Data are shown as mean±SEM with n = 3–7 mice per group (A) and with n = 3–4 mice per group (B).
(TIF)

S3 Fig Effects of adult α-GC on RSV re-infection in adult mice. 7-day-old mice were administered RSV and at 6 weeks age the mice were infected vehicle, α-GC, RSV or α-GC with RSV. Four days after infection, cells were isolated from BAL fluids. Total cell count and the absolute number of eosinophils was determined. Data are mean±SEM with n = 5–6 mice per group.
(TIF)

S4 Fig Effects of α-GC on infiltrated neutrophils during RSV infection. Seven-day-old mice were administered vehicle, α-GC, RSV, or α-GC with RSV. At 8 weeks of age, the mice were infected with RSV or α-GC with RSV. Four days after infection, cells were isolated from BAL fluids. The total cell count and absolute number of neutrophils were determined. Data are expressed as mean ± SEM, with n = 3–5 mice per group.
(TIF)

## Abbreviations

AHR: airway hyperresponsiveness
α-GC: α-galactosylceramide
BAL: bronchoalveolar lavage
CD1d KO: CD1d knockout
FBS: fetal bovine serum
H&E: hematoxylin and eosin
IFN-γa: interferon-γa
IL: interleukin
i.n.: intranasa
PAS: periodic acid–Schiff
MEM: minimum essential media
NKT: natural killer T
iNKT: invariant natural killer T
RSV: respiratory syncytial virus
