# Supplementary figures and images for: Natural killer T cell sensitization during neonatal respiratory syncytial virus infection induces eosinophilic lung disease in re-infected adult mice
Source: PLoS One. 2017 Jun 1;12(6):e0176940. doi: 10.1371/journal.pone.0176940 (PMC5453428; doi:10.1371/journal.pone.0176940)

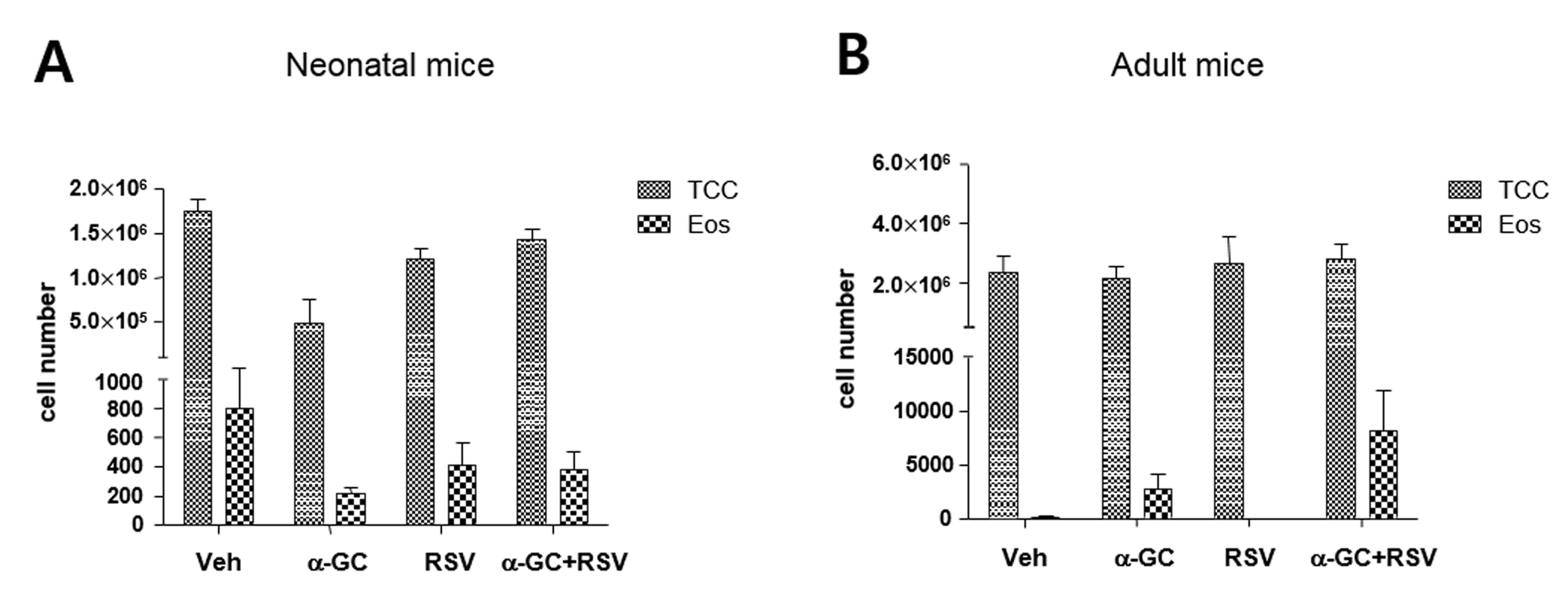

Supplement: S1 Fig — Mice were administered vehicle, α-GC, RSV, or α-GC+RSV via intranasal route when they were 7 days old (neonatal mice) (A) and 6 weeks old (adult mice) (B). 4 days after administration, cells were isolated from BAL fluids and stained with fluorescence-labeled anti-CD45, CD11c, and Siglec-F antibodies. Total cell count and the absolute number of eosinophils, namely CD11c-SiglecF+ cells, was shown. Data are shown as mean±SEM with n = 6–8 mice per group using pooled samples from 3 neonatal mice and representative of 4 independent experiments (A). Data are shown as mean±SEM with n = 4–5 mice per group (individual) and representative of 2 independent experiments (B). (TIF) [file pone.0176940.s001.tif]

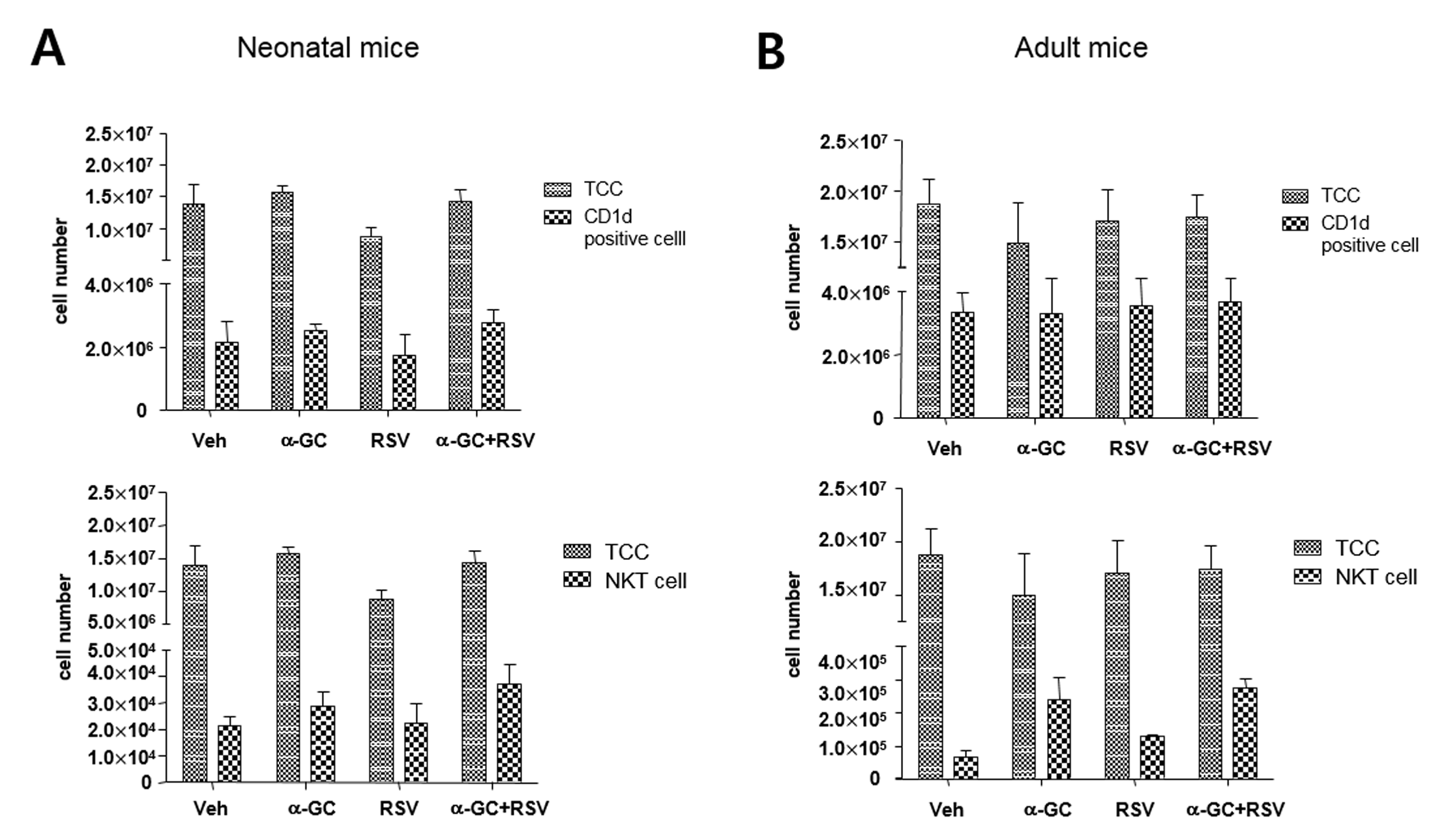

Supplement: S2 Fig — (A) Mice were infected with vehicle, α-GC, RSV, or α-GC+RSV via intranasal route when they were 7 days old (neonatal mice) (A) and 6 weeks old (adult mice) (B). After 4days, lungs were harvested and stained with fluorescence-labeled anti-CD1d, CD3 and CD49b+ antibodies. Total cell count and the absolute number of CD1d positive cell, or NKT cells (CD3+ CD49b+) were shown. Data are shown as mean±SEM with n = 3–7 mice per group (A) and with n = 3–4 mice per group (B). (TIF) [file pone.0176940.s002.tif]

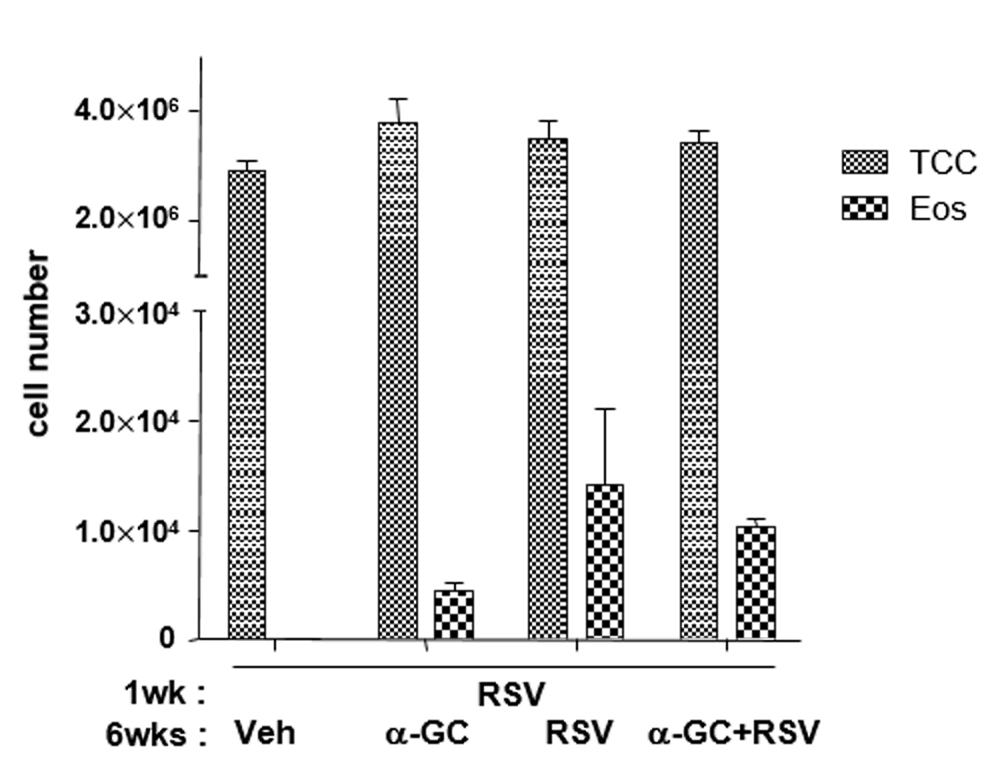

Supplement: S3 Fig — 7-day-old mice were administered RSV and at 6 weeks age the mice were infected vehicle, α-GC, RSV or α-GC with RSV. Four days after infection, cells were isolated from BAL fluids. Total cell count and the absolute number of eosinophils was determined. Data are mean±SEM with n = 5–6 mice per group. (TIF) [file pone.0176940.s003.tif]

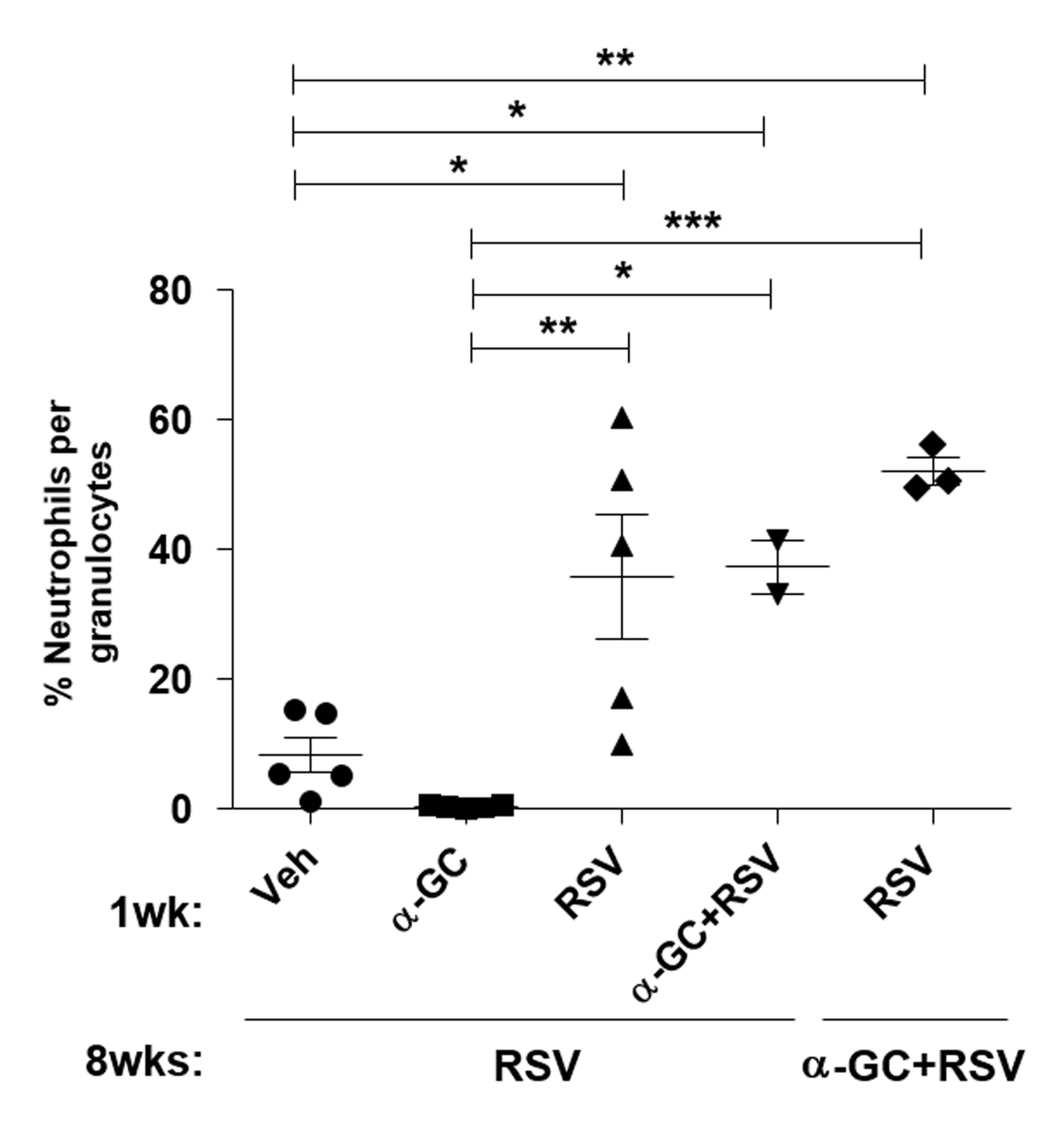

Supplement: S4 Fig — Seven-day-old mice were administered vehicle, α-GC, RSV, or α-GC with RSV. At 8 weeks of age, the mice were infected with RSV or α-GC with RSV. Four days after infection, cells were isolated from BAL fluids. The total cell count and absolute number of neutrophils were determined. Data are expressed as mean ± SEM, with n = 3–5 mice per group. (TIF) [file pone.0176940.s004.tif]
